# ECG Abnormalities and Biomarkers Enable Rapid Risk Stratification in Normotensive Patients With Acute Pulmonary Embolism

**DOI:** 10.1111/crj.70060

**Published:** 2025-06-19

**Authors:** Siqi Jiao, Ying Liu, Haoming He, Qing Li, Zhe Wang, Yinong Chen, Longyang Zhu, Shuwen Zheng, Furong Yang, Zhenguo Zhai, Yihong Sun

**Affiliations:** ^1^ Peking University Health Science Center China‐Japan Friendship Hospital Beijing China; ^2^ Beijing Ditan Hospital Capital Medical University Beijing China; ^3^ Department of Cardiology, China‐Japan Friendship Hospital Chinese Academy of Medical Sciences and Peking Union Medical College Beijing China; ^4^ School of Traditional Chinese Medicine Beijing University of Chinese Medicine Beijing China; ^5^ Department of Pulmonary and Critical Care Medicine, Center of Respiratory Medicine China‐Japan Friendship Hospital Beijing China; ^6^ Institute of Respiratory Medicine Chinese Academy of Medical Sciences Beijing China; ^7^ National Clinical Research Center for Respiratory Diseases Beijing China; ^8^ Department of Cardiology, Beijing Anzhen Hospital Capital Medical University, Beijing Institute of Heart Lung and Blood Vessel Diseases Beijing China

**Keywords:** acute pulmonary embolism, in‐hospital adverse events, low to moderate risk, prognosis

## Abstract

**Background:**

The patients with suspected pulmonary embolism (PE) were usually screened using electrocardiogram (ECG) and blood panel of D‐dimer, troponin, and blood gas analysis in the emergency.

**Objectives:**

This study aimed to explore a rapid risk model to predict in‐hospital adverse events for normotensive PE patients.

**Methods:**

Patients with acute PE having normal blood pressure on appearance were retrospectively enrolled at China‐Japan Friendship Hospital from January 2017 to February 2020. The in‐hospital adverse events were defined as death and clinical deterioration during hospitalization. The risk model for in‐hospital adverse events was generated by multivariate regression analysis. The discrimination ability of the model was compared with PESI, Bova, and FAST risk score, and evaluated by the receiver operating characteristic curve (ROC), net reclassification improvement (NRI), and integrated discrimination improvement index (IDI).

**Results:**

Of the 213 patients, 35 (16.4%) experienced in‐hospital adverse events,y including 15 deaths. The average age was 69 ± 19 years, and 118 (44.6%) were females. Multiple logistic regression analysis showed that independent risk factors associated with in‐hospital adverse events were low QRS voltage in ECG (OR: 5.321; 95% CI: 1.608–7.310), positive age‐adjusted D‐dimer (OR: 2.061; 95% CI: 0.622–6.836), positive troponin (OR: 3.504; 95% CI: 1.744–8.259), and PaO_2_/FiO_2_ < 300 (OR: 3.268; 95% CI: 0.978–5.260). The ROC analysis showed that the AUC of the new model (0.847, 95% CI: 0.786–0.901) was better than the PESI score (0.628, 95% CI: 0.509–0.769), the Bova score (0.701, 95% CI: 0.594–0.808), and the FAST score (0.775 95% CI: 0.690–0.859).

**Conclusion:**

ECG abnormalities and biomarkers on admission may provide a rapid and effective approach to identify patients with poor prognoses during hospitalization.

## Graphical abstract

One ECG abnormality and the elevation of three biomarkers on admission may provide a rapid and effective approach to identify patients with poor prognoses during hospitalization.

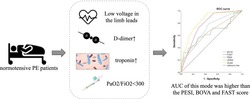

**Abbreviations**
AUC: area under the curve
BNP: brain natriuretic peptide
CI: confidence interval
cTN: cardiac troponin
CTPA: computed tomography pulmonary angiography
DBP: diastolic blood pressure
D‐D: D‐dimer
DVT: deep venous thrombosis
ECG: electrocardiogram
IDI: integrated discrimination improvement index
LV: left ventricle
NRI: net reclassification improvement
NT‐proBNP: N‐terminal pro brain natriuretic peptide
OR: odds ratio
PE: pulmonary embolism
RBBB: right bundle branch block
ROC: receiver operating characteristic
RV: right ventricle
SBP: systolic blood pressure
SE: standard error
SPAP: pulmonary artery systolic pressure
STD: ST segment depression
STE: ST segment elevation
TAPSE: tricuspid annular plane systolic excursion
TWI: T wave inversion

## 1 Introduction

Pulmonary embolism (PE) is the third most common cause of death in hospitalized patients [1]. The severity of PE varies widely with early mortality ranging from < 1% in stable patients to more than 50% in hemodynamic instable patients [2]. Both Pulmonary Embolism Severity Index (PESI) [3] and its simplified version, the simplified PESI (sPESI) [4], are widely accepted as risk stratification tools for 30‐day mortality. These scoring systems are useful to identify low‐risk patients appropriate for an outpatient setting, but not to predict high risk patients for advanced therapy. Several studies have shown that more than 50% of patients with acute PE are hemodynamically stable on admission but have a high risk of death according to clinical models [5, 6]. The 2019 European Society of Cardiology (ESC) guidelines recommended employing PESI or sPESI, imaging signs of right ventricle (RV) dysfunction, troponin, and N‐terminal pro‐brain natriuretic peptide (NT‐proBNP) to classify patients into low, intermediate–low, and intermediate–high risk [7]. Given the limitation of the PESI and sPESI score, risk models to optimize and simplify risk assessment have been developed, such as the FAST score [8] and the Bova score [9]. They are easier to calculate and have been validated in prospective cohort studies [5, 10]. However, there remains controversy about the optimal risk stratification strategy for prognosis in Chinese patients with PE [5, 11, 12, 13].

Electrocardiogram (ECG) has poor sensitivity and specificity for diagnosing APE, but it is still one of the first procedures performed on appearance, mostly in patients who present with chest pain or dyspnea. It was reported that both depolarization and repolarization abnormalities are observed significantly more frequently in high‐risk APE patients. Besides, the combination blood test panel of d‐dimer, troponin, and natriuretic peptide was also used for patients who present to the emergency department with symptoms suggesting APE. The aim of our study was to explore a simple and fast prognostic model to predict in‐hospital adverse events in PE patients with normal blood pressure on appearance. We also compared the performance of our risk model with the PESI, FAST, and Bova scores.

## 2 Methods

### 2.1 Study Design and Participants

Thisretrospective cohort study included normotensive patients (defined as a SBP > 90 mmHg) hospitalized for acute PE in China‐Japan Friendship Hospital from January 2017 to February 2020. Only adult patients with objectively confirmed PE and no reperfusion procedures at the time of PE diagnosis were enrolled. The diagnosis of PE required to be confirmed with computed tomographic pulmonary angiography (CTPA) or scintigraphic ventilation‐perfusion (V/Q) scan revealing high probability of PE. Exclusion criteria were chronic thromboembolic pulmonary hypertension (CTEPH), acute coronary syndrome, and a history of pacemaker implantation.

### 2.2 Data Collection

Clinical information was collected from hospital records or electronic case report form in a web‐based database system. Patient characteristics included demographic information, medical history, signs and symptoms on admission, laboratory tests, and images. The parameters included age, sex, presenting symptoms (dyspnea, chest pain, cough, syncope, unilateral lower limb swelling and pain, and hemoptysis), blood pressure, heart rate, respiratory rate, oxyhemoglobin saturation, cardiogenic shock, or cardiopulmonary resuscitation. Laboratory panel on admission was comprised of D‐dimer (D‐D), troponin I (cTNI) or troponin T (cTNT), B‐type natriuretic peptide (BNP) or NT‐proBNP, and arterial blood gas analysis. Troponin was defined as elevated if cTNI > 0.4 ng/mL or cTNT > 0.014 ng/mL, according to the biochemical tests used in our institution. NT‐ProBNP > 600 pg/mL and BNP > 100 pg/mL were considered as abnormal, and these values were used as cut‐off values according to the ESC guidelines [14]. The RV dysfunction was determined by transthoracic echocardiography or CTPA and defined as positive if at least one of the following criteria is met, (1) tricuspid annular plane systolic excursion (TAPSE) < 16 mm; or (2) RV/LV > 1.0; or (3) sPAP (systolic pulmonary artery pressure) > 50 mmHg. We also collected the following parameters based on the first ECG: S1Q3T3, incomplete and complete RBBB, Qr pattern in lead V1, low QRS voltage in limb leads, ST‐segment elevation (STE) or ST‐segment depression, T‐wave inversion in leads II/III/aVF, and T‐wave inversion in leads V1‐V3/V4. The low QRS voltage in limb leads was defined as peak‐to‐nadir QRS voltage less than 5 mm (0.5 mV) in all limb leads and less than 10 mm (1.0 mV) in all precordial leads.

### 2.3 Scores and Algorithms Assessment

The PESI score [3] ranges from 0 to > 125 points, based on the following criteria: age in years, male sex (10 points), cancer (30 points), chronic heart failure (10 points), chronic pulmonary disease (10 points), pulse rate ≥ 110 bpm (20 points), systolic blood pressure < 100 mmHg (30 points), respiratory rate > 30 breaths per min (20 points), temperature < 36°C (20 points), altered mental status (60 points), and arterial oxyhemoglobin saturation < 90% (20 points). The sPESI [4] is based on six clinical characteristics, attributing 1 point for each: age, cancer, chronic heart failure or pulmonary disease, pulse rate ≥ 110 bpm, systolic blood pressure (BP) < 100 mmHg, and arterial oxyhemoglobin saturation < 90%. Low‐risk classification is defined in the PESI as ≤ 85 points (Classes I and II) and in the sPESI as 0 points.

The Bova score [9] includes four parameters (1 point for each): elevated cardiac troponin, RV dysfunction (documented on transthoracic echocardiogram or CTPA), heart rate ≥ 110 bpm, and systolic BP 90–100 mmHg. Low‐risk classification is defined as ≤ 2 points.

The modified FAST score [15] includes 3 parameters: elevated cardiac troponin (1.5 point), syncope (1.5 point), and heart rate ≥ 100 bpm (2 points). Based on this, the patients were split into low risk (< 3 points) and intermediate‐high risk (≥ 3 points) (Table S1).

### 2.4 The Definition of Clinical Outcomes

The clinical outcomes were in‐hospital adverse events, defined as in‐hospital death and clinical deterioration. Clinical deterioration included hypotension with SBP < 90 mmHg for ≥ 15 min or need for catecholamine administration, receiving thrombolysis therapy, mechanical ventilation, or cardiopulmonary resuscitation during hospitalization.

### 2.5 Statistical Analysis

Continuous variables were expressed as mean and standard deviation or median and interquartile range (IQR), according to their distribution. Categorical variables were expressed as numbers and percentages. For the comparisons between continuous variables, the independent sample *t*‐test and Mann–Whitney *U*‐test were used. Comparisons between proportions were made using the χ^2^ test or Fisher's exact test. An alpha error of less than 5% was assumed to establish statistical significance. Candidate variables associated with in‐hospital adverse events on univariate analysis (*p* < 0.05) were included as potential covariates in a multiple logistic regression model to assess independent predictor factors.

The area under the receiver operating characteristic (ROC) curve (AUC) was calculated to assess the prognostic accuracy and discriminative ability of our model compared to PESI score, FAST score, and Bova score. The Youden index was used to determine the cut‐off point with the highest sensitivity and specificity. To quantify the predictive power and the added predictive ability of our model, Net Reclassification Improvement (NRI) and integrated discrimination improvement index (IDI) were calculated. Subsequently, the contribution of biomarkers to discriminate adverse events was evaluated. Statistical analysis was performed using the SPSS (IBM SPSS Statistics for Windows, version 225.0: IBM corporation) and R 4.1.2 (mada, metafor).

## 3 Results

### 3.1 Baseline Characteristic

**FIGURE 1:**
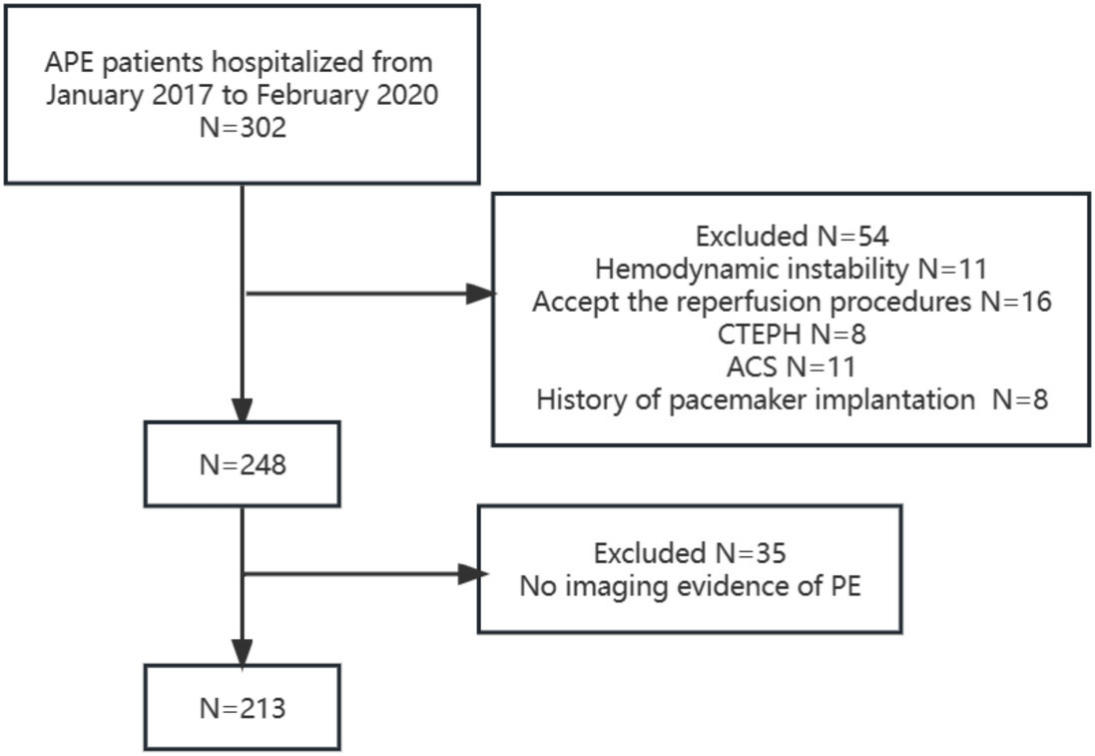
Flow diagram of patient enrolment . ACS, acute coronary syndrome; CTEPH, chronic thromboembolic pulmonary hypertension; PE, pulmonary embolism.

Three hundred two patients hospitalized for acute PE were screened and 213 patients were included in the analysis (Figure 1). The mean age of the patients was 69 ± 19 years, and 44.6% were men. The diagnose of PE was confirmed by CTPA in 179 (84.0%) patients and by V/Q scan in 34 (16.0%) patients. Baseline characteristic was shown in Table 1. Compared to the patients without in‐hospital adverse events, patients with in‐hospital adverse events had higher frequencies of syncope (40% vs. 12.4%, *p* < 0.001), lower diastolic blood pressure (69 ± 16 vs. 77 ± 13, *p* = 0.009), higher heart rate (95 ± 24 vs. 85 ± 17, *p* = 0.031), and higher level of D‐dimer. They also more likely to have positive troponin and elevated BNP/NT‐proBNP.

**TABLE 1:** Clinical characteristics.

Characteristics	All (*n* = 213)	Patients with in‐hospital adverse events (*n* = 35)	Patients without in‐hospital adverse events (*n* = 178)	*p*
Age (years)	69 ± 19	66 ± 21	69 ± 18	0.444
Male sex	95 (44.6%)	14 (40.0%)	81 (45.5%)	0.686
Former smoking	33 (15.5%)	4 (11.4%)	29 (16.3%)	0.711
Smoking	24 (11.3%)	4 (11.4%)	20 (11.2%)	1
Symptoms on admission				
Dyspnea	164 (77.0%)	31 (88.6%)	133 (74.7%)	0.075
Chest pain	75 (35.2%)	6 (17.1%)	69 (38.8%)	0.014
Syncope	36 (16.9%)	14 (40.0%)	22 (12.4%)	< 0.001
Hemoptysis	21 (9.9%)	4 (11.4%)	17 (9.6%)	0.984
Comorbidities				
DVT	107 (50.2%)	16 (45.7%)	91 (51.1%)	0.734
Liver dysfunction	31 (14.6%)	6 (17.1%)	25 (14.0%)	0.635
Renal dysfunction	11 (5.2%)	4 (11.4%)	7 (3.9%)	0.175
Ischemic stroke	40 (18.8%)	8 (22.9%)	32 (18.0%)	0.562
CHF	36 (16.9%)	9 (25.7%)	27 (15.2%)	0.128
Malignancy	36 (16.9%)	9 (25.7%)	27 (15.2%)	0.128
Chronic lung disease	27 (12.7%)	5 (14.3%)	22 (12.4%)	0.972
Atrial fibrillation/atrial flutter	17 (8.0%)	5 (14.3%)	12 (6.7%)	0.244
SBP (mmHg)	129 ± 18	125 ± 20	130 ± 17	0.144
DBP (mmHg)	76 ± 13	69 ± 16	77 ± 13	0.009
Heart rate (b.p.m)	87 ± 18	95 ± 24	85 ± 17	0.031
Respiratory rate (beats/min)	20 ± 2	21 ± 2	20 ± 2	0.087
SO_2_ (%)	96 ± 4	96 ± 4	96 ± 3	0.508
Electrocardiogram				
Tachycardia	45 (21.1%)	14 (40.0%)	31 (17.4%)	0.003
S1Q3T3	53 (24.9%)	13 (37.1%)	40 (22.5%)	0.066
RBBB	20 (9.4%)	5 (14.3%)	15 (8.4%)	0.442
QR pattern in V1	21 (9.8%)	6 (17.1%)	15 (8.4%)	0.204
Low QRS voltages	31 (14.6%)	10 (28.6%)	21 (11.8%)	0.010
STE in V1–V3/V4	13 (6.1%)	1 (2.9%)	12 (6.7%)	0.623
STE in aVR	18 (8.5%)	5 (14.3%)	13 (7.3%)	0.305
STD in V4–V6	13 (6.1%)	5 (14.3%)	8 (4.5%)	0.068
TWI in II/III/aVF	82 (38.5%)	15 (42.8%)	67 (37.6%)	0.562
TWI in V1–V3/V4	75 (35.2%)	16 (45.7%)	59 (33.1%)	0.155
AF	4 (1.8%)	0	4 (2.2%)	0.227
Laboratory test				
Age‐adjusted D‐D+	60 (28.1%)	20 (57.1%)	40 (22.5%)	< 0.001
cTn+	111 (52.1%)	28 (80.0%)	83 (46.6%)	0.001
BNP/nt+	104 (48.8%)	23 (65.7%)	81 (45.5%)	0.039
Biomarker+	160 (75.1%)	32 (91.4%)	128 (72.0%)	0.015
PaO_2_/FiO_2_+	81 (38.0%)	23 (65.7%)	58 (32.6%)	0.003
RV dysfunction	126 (59.2%)	24 (68.6%)	102 (57.3%)	0.440
TAPSE (mm)	18.24 ± 5.11	19.86 ± 4.74	18.66 ± 4.59	0.447
SPAP (mmHg)	49.22 ± 24.36	46.78 ± 13.56	38.96 ± 5.37	0.126
RV dilation	45 (20.5%)	7 (31.8%)	38 (26.6%)	0.607

Abbreviations: BNP, brain natriuretic peptide; cTn, cardiac troponin; DBP, diastolic blood pressure; D‐D, D‐dimer; DVT, deep venous thrombosis; RBBB, right bundle branch block; RV, right ventricle; SBP, systolic blood pressure; SPAP: pulmonary artery systolic pressure; STD, ST segment depression; STE, ST segment elevation; TAPSE, tricuspid annular plane systolic excursion; TWI, T wave inversion.

Patients with in‐hospital adverse events were more likely to have tachycardia, S1Q3T3, low limb voltages, and ST depression in V4–V6 as shown in 12‐lead ECG. However, there was no difference on the incidence of RV dysfunction between groups (*p* = 0.440).

### 3.2 Distribution of Patients With Low and High Risk Based on the PESI, sPESI, Bova, and FAST Score

**FIGURE 2:**
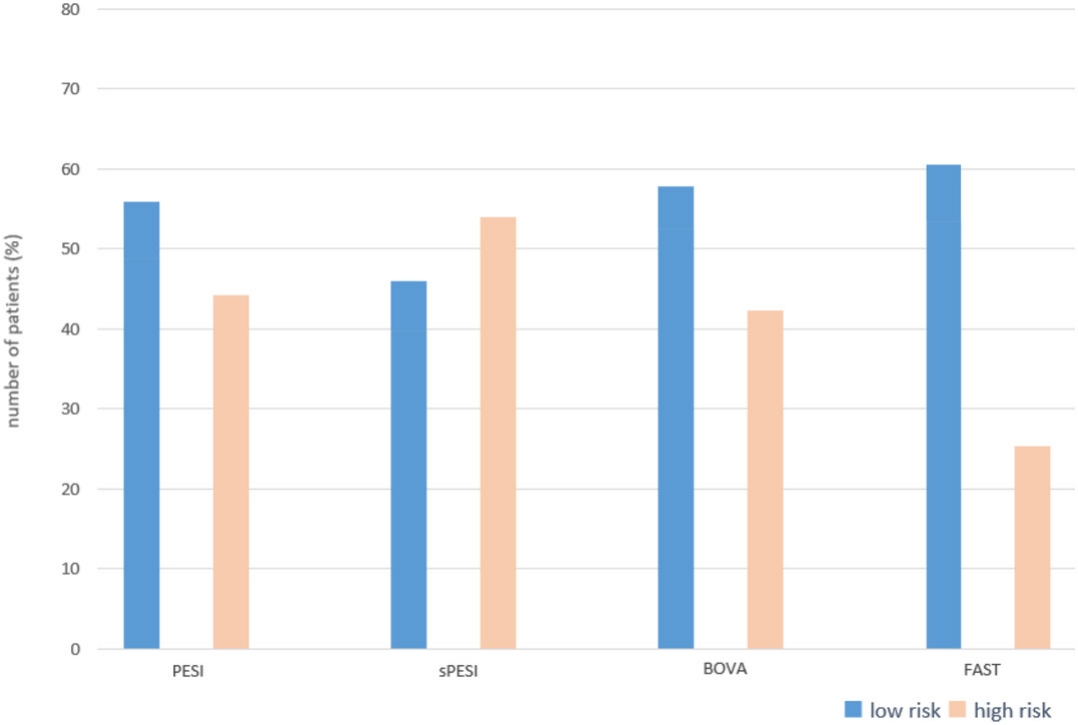
Distribution of the proportion of the patients with high risk based on risk stratification. All the risk scores stratify patients as low risk and high risk. The risk scores are defined in Table S1.

The proportion of the patients categorized as high risk was 44.1% (94) and 54.0% (115) using the PESI and sPESI score, respectively. Overall, 42.3% (90) of the patients were belonged to high risk based on Bova score, and the modified FAST score classified 39.4% (84) patients as high risk (Figure 2)

### 3.3 Predictors for In‐Hospital Adverse Events

Overall, the incidence of in‐hospital adverse events was 16.4% (35), including 15 deaths and 20 patients with clinical deterioration. Univariate logistic regression analysis showed that HR ≥ 110 beats/min, tachycardia, low limb voltages, positive age‐adjusted D‐dimer, positive troponin, elevated BNP levels, and PaO_2_/FiO_2_ < 300 were significantly associated with in‐hospital adverse events (Table 2). Multivariate logistic regression showed that the independent risk factors for in‐hospital adverse event were low QRS voltage in ECG (OR: 5.321; 95% CI: 1.608–7.310), positive age‐adjusted D‐dimer (OR: 2.061; 95% CI: 0.622–6.836), positive troponin (OR: 3.504; 95% CI: 1.744–8.259), and PaO_2_/FiO_2_ < 300 (OR: 3.268; 95% CI: 0.978–5.260).

**TABLE 2:** Univariate and multivariate analyses of variables associated with the in‐hospital adverse events.

Variables	Univariate	OR (95% CI)	Multivariate final model	
	*p*		*p*	OR (95% CI)
Heart rate ≥ 110 bpm	0.007	3.347 (1.397–8.023)	0.196	2.016 (0.696–5.840)
Tachycardia	0.004	3.079 (1.444–6.562)	0.492	2.402 (0.283–9.422)
Low QRS voltages	0.013	2.99 (1.261–7.090)	0.033	5.321 (1.608–7.310)
Age‐adjusted D‐D+	< 0.01	4.176 (2.001–8.715)	0.037	2.061 (0.622–6.836)
cTn+	0.001	4.434 (1.839–10.687)	0.005	3.504 (1.744–8.259)
BNP+	0.042	2.201 (1.030–4.700)	0.975	0.984 (0.358–2.702)
PaO_2_/FiO_2_ < 300	0.004	3.193 (1.441–7.077)	0.031	3.268 (0.978–5.260)
RV dysfunction	0.249	1.835 (0.654–5.150)		Not included
RV dilation	0.608	1.289 (0.488–3.404)		Not included

Abbreviations: cTn, cardiac troponin; D‐D, D‐dimer; OR, odds ratio; RV, right ventricle.

The four variables independently associated with in‐hospital adverse events were assigned points for the risk score according to the regression coefficients obtained as follows: low QRS voltages in ECG (5 points), positive age‐adjusted D‐dimer (2 points), positive troponin (3 points), and PaO_2_/FiO_2_ < 300 (3 points) (Table S2). The cut‐off of our model ≥ 8 scores revealed a 70.0% sensitivity and 83.9% specificity for the prediction of in‐hospital adverse events (Table 3).

**TABLE 3:** AUC of ROC curves, sensitivity, and specificity of MOEDL, PESI, sPESI, BOVA, and FAST.

	AUC (95% CI)	Sensitivity (%)	Specificity (%)
MODEL	0.847 (0.786–0.901)	76.0	86.9
PESI	0.639 (0.509–0.769)	57.1	58.4
sPESI	0.638 (0.517–0.758)	62.9	47.8
BOVA	0.701 (0.594–0.808)	68.6	62.9
FAST	0.775 (0.690–0.859)	57.1	77.0

Abbreviations: AUC, area under the curve; CI, confidence interval.

### 3.4 Comparison of Risk Prediction Models for In‐Hospital Adverse Events

**FIGURE 3:**
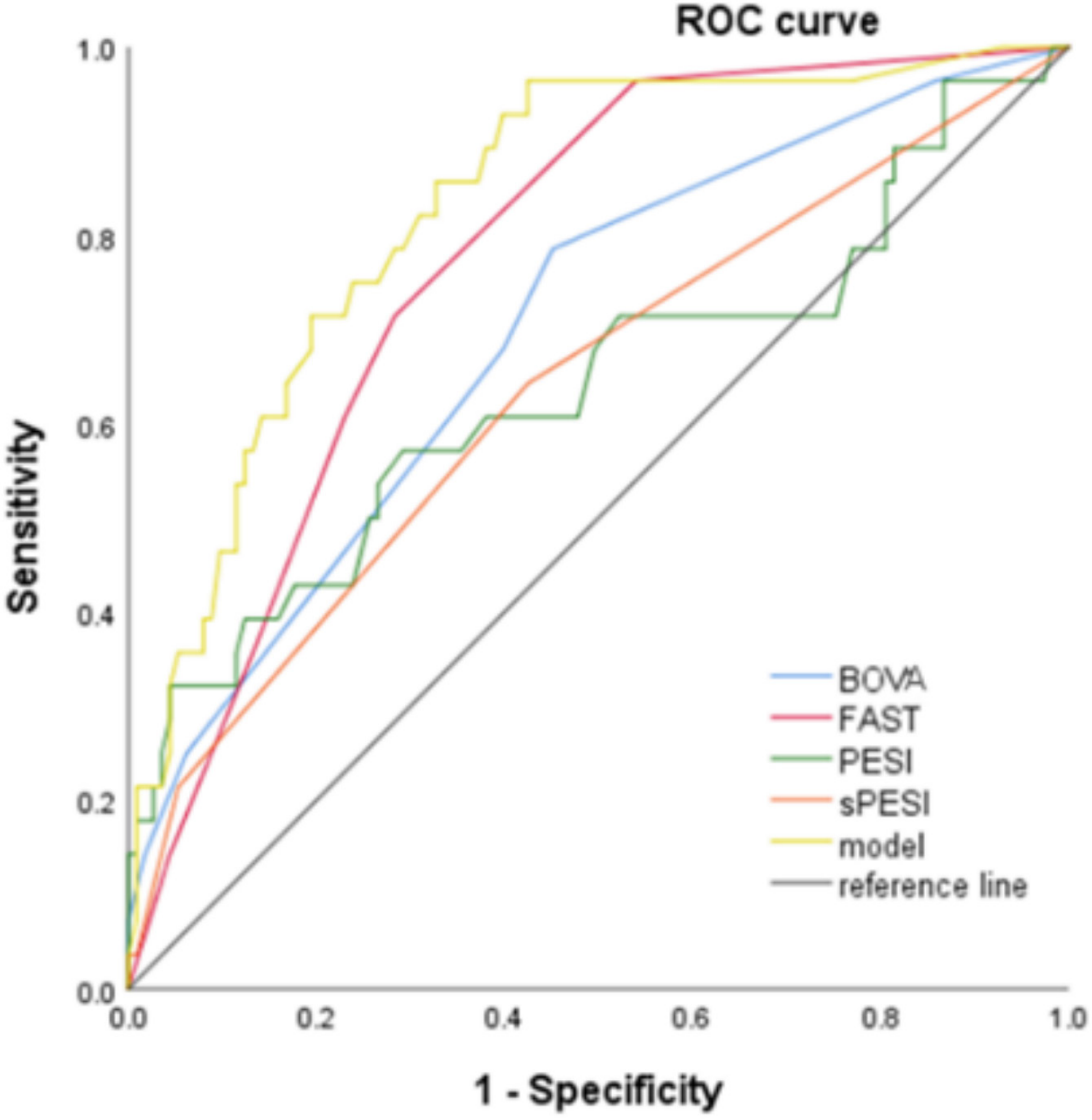
Receiver operating characteristics (ROC) analysis of risk assessment strategies with regard to in‐hospital adverse outcomes. Model: a total point score for a given patient is obtained by summing the points: low QRS voltages in ECG (5 points), higher D‐D (per 2 mg/L) (1 points), positive troponin (3 points), and PaO_2_/FiO_2_ < 300 (3 points).

Figure 3 depicts the ROC curves of Bova score, FAST score, PESI score, sPESI score, and our model. The predictive ability of our model (0.847) is better than the Bova score (0.701), the FAST score (0.775), the sPESI (0.638), and the PESI score (0.639), respectively (Table 3). Remarkable improvements in reclassification and discrimination were achieved by our model when compared with the PESI (NRI = 39.8%, IDI = 24.2%), the Bova (NRI = 25.2%, IDI = 25.6%), and the FAST score (NRI = 26.1%, IDI = 18.2%) (Table 4). However, it is worth noting that although our model has better absolute values, no significant improvement was found when compared with FAST score.

**TABLE 4:** The NRI and IDI were used to assess reclassification performance and improvement in discrimination of our novel prediction model.

	NRI	*p*	IDI	*p*
Our model vs. sPESI	0.423	0.001	0.254	0.008
Our model vs. PESI	0.398	0.003	0.242	0.009
Our model vs. BOVA	0.252	0.032	0.256	0.032
Our model vs. FAST	0.261	0.053	0.182	0.072

Abbreviations: IDI, integrated discrimination improvement index; NRI, net reclassification improvement.

## 4 Discussion

In this single‐center retrospective study of patients with acute PE, we had several findings. First, the incidence of in‐hospital adverse events was high (16.4%) in this cohort of acute PE patients with normal blood pressure. Second, we found that one simple ECG abnormal and three biomarkers rapidly available in the emergency could predict in‐hospital adverse events, which included D‐dimer, troponin, and PaO_2_/FiO_2_. It showed better predictive performance compared to the PESI, sPESI, Bova, and FAST scores. This may offer a simple, readily available, and fast approach to immediate risk assessment for patients with acute PE.

In our study, the incidence of in‐hospital adverse events is 16.4%, and the mortality is 7.0% in normotensive PE patients. The overall 30‐day mortality rates of the PE patients, irrespective of the severity, were 9.2% and 7.8% in PESI and sPESI derivation samples, respectively [16, 17]. An Italian registry reported that the in‐hospital mortality rate was 31.8% in hemodynamically unstable patients, but a much lower incidence of death in hemodynamically stable patients (3.4%) [18]. Bova et al. reported that the 30‐day mortality was 6.1% in normotensive patients with PE [9]. Lukas et al. found that the rate of 30‐day adverse events was 6.4%, and the mortality was 3.6% in the normotensive PE patients [5]. Compared to the previous studies, the patients enrolled in this study had a higher prevalence of chronic heart failure and arterial vascular disease at baseline, which may lead to higher mortality. Therefore, risk stratification for patients with low to moderate risk could be helpful to identify those with poor prognosis and apply the appropriate treatment strategy.

ECG was routinely performed for patients suspected of PE. Although there are no specific ECG findings indicative of PE, the potential use for risk stratification in the management of PE has been suggested in a previous study [19]. Several ECG features have been proven to be associated with a negative outcome in patients with acute PE, including tachycardia, atrial fibrillation, S1Q3T3, RBBB, T‐wave inversion, ST segment abnormality, and so on [20, 21]. We found that low limb lead QRS voltage in ECG was strongly associated with the risk of in‐hospital adverse outcomes, along with previous studies. Prior research similarly indicates that low QRS voltage is related to APE and its severity [22, 23]. The mechanisms that determine QRS amplitude are hotly debated. Kukla et al. had written extensively on the topic and showed that low QRS voltage is usually caused by conditions that either impair voltage generation or alter voltage transmission from the myocardium to the skin electrodes [24]. Pathologic mechanisms underlying low QRS voltage in PE seem to be more complex, and some possible explanations can be speculated. Given that sudden pressure overload in the pulmonary circulation may lead to acute RV dysfunction in PE patients, low QRS voltages may likely reflect RV dysfunction and the resulting peripheral edema [25]. On the other hand, low QRS voltages may be the consequence of a significant loss of the “electrically active” myocardium caused by ischemia and hypoxia in PE [26]. Finally, pulmonary edema [27, 28] and pericardial effusion [29, 30], which have been shown to occur in a significant proportion of PE patients, may lead to low QRS voltages, possibly through an electric signal attenuation effect. Moreover, several investigations have previously demonstrated the prognostic role of dynamic serial ECG in PE patients [31, 32, 33]. Marco et al. analyzed the ECG modifications of 687 individuals with acute PE enrolled in the IPER and identified that persistence of RBBB, negative T waves, and qR pattern in V1 lead at day 3 of hospitalization are independent prognostic factors of death within 30 days in high‐risk acute PE patients [34]. The electrocardiographic changes in PE may take a role and give further information in the prognostic assessment and in predicting adverse clinical outcomes [35]. Regrettably, in our study, we only collected the parameters of the patient's initial ECG without collecting them repeatedly. However, our prediction rule may represent a non‐invasive, widely available, and low‐cost method which allows for the rapid and precise risk stratification of PE patients.

D‐dimer as a degradation product of cross‐linked fibrin has a high sensitivity and negative predictive value for acute PE. Normal D‐dimer values exclude an acute PE in hemodynamically stable patients with high probability. Besides, computed tomography (CT) studies suggest that higher D‐dimer levels are associated with significantly higher clot burden in pulmonary arteries [36, 37, 38]. Furthermore, D‐dimer level was also associated with hypoxemia, myocardial injury, and the severity of RV function determined by imaging [39, 40]. An International prospective RIETE study confirmed that plasma concentration of D‐dimer is an independent factor associated with all‐cause and PE‐related death [41]. A small study of non‐massive PE demonstrated that D‐dimer lower than 2000 μg/L had a 98% NPV for adverse outcomes during the 3‐month follow‐up period [42]. Therefore, the high level of D‐dimer is an indicator of worse hospital outcomes in patients with acute PE [43].

Hypoxemia is one of the parameters in the calculation of PESI and sPESI score, but not in Bova and FAST score. Blood gas has been routinely measured in the diagnostic algorithm of acute PE. Yan et al. found that the PaO_2_/FiO_2_ ratio lower than 265 was an additional predictor of in‐hospital mortality in a Chinese cohort of patients with acute PE [44]. We extended this finding in PE patients with low to moderate risk. In our study, PaO_2_/FiO_2_ < 300 is associated with a higher incidence of in‐hospital adverse events.

RV dysfunction is an important predictor of the severity of acute PE, which could be assessed by imaging and biomarkers of myocardial injury and right heart failure [7]. A meta‐analysis including 7536 patients from 22 studies suggested the presence of RV dysfunction as indicated by imaging findings or laboratory markers on admission was associated with early mortality in patients who are identified as “low‐risk” by PESI/sPESI or Hestia scores [45]. We confirmed that both elevated troponin and BNP wwere associated with in‐hospital adverse outcomes in acute PE patients [46, 47], consistent with prior studies [48, 49, 50]. Elevated troponin is also a risk factor in both Bova and the modified FAST score. However, RV dysfunction identified by imaging was not independently associated with in‐hospital adverse outcomes in our study. There were several explanations for this. First, the definition of RV dysfunction in our study is different from previouse study. We defined RV dysfunction as RV/LV > 1.0, elevated sPAP, or abnormal tricuspid annular plane systolic excursion (TAPSE < 15 mm), while some studies also had hypokinesis of the RV free wall or abnormal motion of the interventricular septum as the criteria for RV dysfunction. Second, the RV dysfunction assessed by imaging is heterogeneous and has proved to be difficult to standardize [51]. Third, the RV dysfunction related to acute PE could resolve rapidly under effective treatment [52]. Therefore, delayed echocardiographic assessment of RV dysfunction may have led to study bias.

### 4.1 Limitations

This study has several limitations that need to be discussed. First, due to the limited sample size, we defined the endpoint as in‐hospital adverse events rather than early mortality. However, this endpoint definition has been widely used in the derivation of multiple PE risk stratification models, such as Bova, FAST, and ROCky [53]. Second, given the single‐center retrospective design of our study, this may lead to selection bias, and thus, multi‐center prospective studies are also needed to evaluate the clinical utility of our model. Third, patients with missing data for the calculation of risk scores were not included in the analysis, which may introduce unmeasured selection bias. Fourth, we did not collect the information on the treatment during hospitalization, such as the dose of medications. However, all the patients received anticoagulants for PE in our study. Another limitation is that various factors such as patient breathing, and the ability of physicians to record and interpret ECGs may affect the accuracy of ECG results. For this reason, all ECGs were performed by a qualified technician and examined by at least two experienced cardiologists who were blinded to all other clinical findings. In case of disagreement a third cardiologist was consulted. Additionally, many patients will have changes occur in their ECG over time, and we did not perform multiple consecutive monitoring of the patient's ECGs. Last, the optimal combination of biomarkers and its cutoff levels remain to be determined. Some biomarkers that have been confirmed their prognostic value in PE, such as lactate [54] and growth differentiation factor‐15(GDF‐15) [55], were not included in our analysis.

## 5 Conclusion

In conclusion, we found that the risk of in‐hospital adverse events was high in normotensive patients with acute PE. The model based on the parameters that are available within 24 hours after presenting to the hospital showed better predictive performance than PESI, Bova, and FAST scores. The significant predictors included low QRS voltage in ECG, elevated D‐dimer (per 2 mg/L), positive troponin, and PaO_2_/FiO_2_ < 300. This risk index could assist physicians to identify the patients with high risk of clinical deterioration during hospitalization. The value of this model should be validated in large cohort in the future.

## Author Contributions

Siqi Jiao, Ying Liu, and Yihong Sun contributed to the conception and design of the study. All authors performed the data extraction and statistical analysis. Siqi Jiao wrote the main manuscripts. All authors have read and approved the final version submitted.

## Ethics Statement

The study protocol was conducted in accordance with the amended Declaration of Helsinki and was approved by the local independent Ethic Committees at China‐Japan Friendship Hospital. All authors approved the manuscript to publish.

## Conflicts of Interest

The authors declare no conflicts of interest.

## Supporting information


**Table S1** Risk scores of acute pulmonary embolism.
**Table S2** Independent predictors of in‐hospital adverse events and point scoring system of the prediction model.

## Data Availability

The data that support the findings of this study are available from the corresponding author upon reasonable request.
