# Supplementary material for: ECG Abnormalities and Biomarkers Enable Rapid Risk Stratification in Normotensive Patients With Acute Pulmonary Embolism
Source: Clin Respir J. 2025 Jun 19;19(6):e70060. doi: 10.1111/crj.70060 (PMC12178209; doi:10.1111/crj.70060)
Supplement: Supplementary file 1 — Table S1 Risk scores of acute pulmonary embolism. Table S2 Independent predictors of in‐hospital adverse events and point scoring system of the prediction model. [file CRJ-19-e70060-s001.docx]

**Table S1 Risk Scores of acute pulmonary embolism.**

| **Score** | **PESI** |  | **sPESI** | **BOVA** |  | | **FAST** |  |
| --- | --- | --- | --- | --- | --- | --- | --- | --- |
| Items (points) | Age, in years | N | 1 | Elevated cTn | | 2 | Elevated cTn | 1.5 |
|  | Male | 10 |  | RV dysfunction (TTE or CT) | | 2 | Syncope | 1.5 |
|  | Cancer | 30 | 1 | HR≥110 | | 1 | HR≥110 | 2 |
|  | Heart failure | 10 |  | SBP 90–100 | | 2 |  |  |
|  | Chronic lung disease | 10 | 1 |  | |  |  |  |
|  | HR ≥ 110 | 20 | 1 |  | |  |  |  |
|  | SBP < 100 | 30 | 1 |  | |  |  |  |
|  | RR > 30 | 20 |  |  | |  |  |  |
|  | Temp < 36°C | 20 |  |  | |  |  |  |
|  | Altered mental status | 60 |  |  | |  |  |  |
|  | SO_2_ < 90% | 20 | 1 |  | |  |  |  |
| Risk classes |  |  |  |  | |  |  |  |
| Low risk | I-II (≤85) |  | 0 | 0-2 | |  | <3 |  |
| High risk | III-V (>85) |  | 1 | ＞2 | |  | ≥3 |  |

**Abbreviations: SBP, systolic blood pressure; cTn, cardiac troponin; RV, right ventricle; RR, respiratory rate; HR, heart rate.**

**Table S2 Independent predictors of in-hospital adverse events and point scoring system of the prediction model.**

| **Variable** | **β coefficients** | | **95% CI** | **Points** |
| --- | --- | --- | --- | --- |
| low QRS voltages in ECG | | 5.321 | 1.608-7.310 | 5 |
| age‐adjusted D-D+ | | 2.061 | 0.622-6.836 | 2 |
| cTn+ | | 3.504 | 1.744-8.259 | 3 |
| PaO2/FiO2<300 | | 3.268 | 0.978-5.260 | 3 |

Abbreviations: CI, confidence interval; ECG, electrocardiogram; D-D, D-dimer; cTn, cardiac troponin.
